# PET/MRI is superior to PET/CT in detecting oesophago and gastric carcinomas: a meta-analysis

**DOI:** 10.1186/s40644-025-00871-3

**Published:** 2025-04-07

**Authors:** Bo Peng, Hui Sun, Jian Hou, Jian-Xing Luo

**Affiliations:** 1 Department of Radiology, Hospital of Chengdu University of Traditional Chinese Medicine, Chengdu, Sichuan China; 2 Department of Clinical Medicine, Chengdu University of Traditional Chinese Medicine, Chengdu, Sichuan China; 3 Department of Infectious Diseases, Hospital of Chengdu University of Traditional Chinese Medicine, Chengdu, Sichuan China

**Keywords:** PET/MRI, PET/CT, Neoplasm staging, Oesophago carcinomas, Gastric carcinomas

## Abstract

**Objectives:**

To compare the accuracy rates of the detection and staging of oesophago and gastric carcinomas between PET/MRI and PET/CT.

**Methods:**

An extensive librarian-led literature search of PubMed, Embase, Web of Science, the Cochrane Central Library, and CNKI was performed and a meta-analysis was done.

**Results:**

Six studies, including 123 participants, were analyzed. PET/MRI had a comparatively high sensitivity in primary lesion detection compared with PET/CT. (RR = 1.14, 95% CI 1.01–1.29, *P* = 0.036).PET/MRI had no significant statistical differences in all aspects of TNM staging compared with PET/CT.

**Conclusions:**

This systematic review confirmed the advantage of PET/MRI in detecting oesophago and gastric carcinomas.Compared with PET/CT, it can reduce unnecessary radiation exposure and can be used in relevant patients without contraindications of MRI.

## Introduction

Oesophago and Gastric carcinomas are the common malignant tumor of the digestive system which has a high morbidity and mortality rate[1, 2]. Early surgery or endoscopic resection is the primary treatment for oesophago and gastric carcinomas, so early diagnosis and accurate staging has a significant impact on the prognosis [3].

Traditional examination methods for digestive system tumors mainly include X-ray barium meal, CT and Endoscopic ultrasound, but their common limitations means that new imaging technologies are needed to improve the delineation of disease extent, the detection of lymph node metastases, and the assessment of treatment response.In recent years, with the update of MRI scanning technology, its application in digestive system tumors has gradually been recognized [4]. Whole-body fully integrated PET/MRI combining with the advantages of PET imaging with MRI has the advantage of superior soft tissue contrast, and it can provide crucial information such as tumour depth and nodal involvement, as well as tumor function and metabolism, which has been widely introduced in the clinical practice in recent years [5]; however, there are still few literatures focus on the application of PET/MRI in the detection and staging of digestive system tumors[6, 7].

This article searched the comparative studies comparing PET/MRI and PET/CT in the detection and staging of oesophago and gastric carcinomas. We discussed the difference in the detection rate of the primary tumor, lymph node metastasis, and the other metastasis. This article aimed to provide a better choice for patients with oesophago and gastric carcinomas in the screening, condition evaluation and treatment effect monitoring, and finally improving of the survival benefit.

## Materials and methods

The protocol of this meta-analysis was registered in PROSPERO (CRD42024598923).

This systematic review was based on the Preferred.

Reporting Items for Systematic Reviews and Meta-analysis (PRISMA) statements.

### Study selections

The related studies were retrieved in the following databases: PubMed, Embase, Web of Science, the Cochrane Central Library, and CNKI from inception to 1st September 2024.For all databases, the search strategy includes the use of the following terms:“PET/MRI”,“PET/CT”,“Oesophago Carcinomas”or“Gastric Carcinomas”.To prevent missed cases, we also increased manual search, manual search strategy only includes the “PET/MRI”,“Oesophago Carcinomas”or“Gastric Carcinomas”.This meta-analysis was in line with the Critical Appraisal Skills Programme Checklist.Data extraction and conformity assessment were conducted by two independent reviewers.The differences among the reviewers were resolved through group discussion.

### Inclusion and exclusion criteria

Two independent reviewers assessed eligibility and reached a consensus by discussing differences with a third investigator.The evaluation was repeated twice. First of all, the title and abstract were preliminarily evaluated, and the full text was evaluated after the potentially qualified study was selected. No reviewers were blinded to the authors of these studies.

### Inclusion criteria

#### (1) Type of study

The analysis included only comparative trials published in fully peer-reviewed journals before 1st September 2024.

#### (2) Language

Only English and Chinese articles were included.

#### (3) Type of intervention

Both two different diagnostic techniques for detection and staging of oesophago and gastric carcinomas.

#### (4) Type of participants

Patients who developed oesophago or gastric carcinomas were the target population for the meta-analysis.

### Exclusion criteria

(1) Non-comparative trials and unpublished studies were excluded.

(2)No final pathological staging results were excluded.

(3)No relevant results(Did not contain all the PET/MRI staging details and PET/CT staging details)were found.

### Data collection

We extracted the following data: first author, year of the study, country of origin, number of participants, age, radiopharmaceuticals, final pathological staging results, PETMRI/CT staging results. Two authors independently extracted and cross-checked all data. The differences were resolved through in-depth discussions with a third reviewer until we reached a consensus.

### Evaluation of quality of evidence

Two independent reviewers blindly evaluated the methodological quality of the selected studies. Differences were discussed among the groups and resolved by a third evaluator. The risk of bias tool suggested by the Cochrane Handbook for Systematic Reviews of Interventions was used to adjudicate the methodological quality of RCTs [8]. The Newcastle-Ottawa Scale was used to assess the methodological quality of non-RCTs [9].

### Statistical analysis

Stata software ver.12 was used to conduct statistical analysis. The Q test and I2 statistic were used to assess the heterogeneity of the detecting effects. Significant heterogeneity was defined as *p* < 0.1 and I2 > 50%, and the random effect model was used, otherwise, the fixed effect model was used. The relative risk and their 95% confidence interval were calculated. Publication bias was assessed qualitatively by Funnel plot, and statistically using Egger’s and Begg’s test. Sensitivity analysis was conducted by excluding a single study and recalculating the pooled estimates. *P* < 0.05 was considered to be significant (*p* values were two-sided).

## Results

### Characteristics of studies

**Fig. 1:**
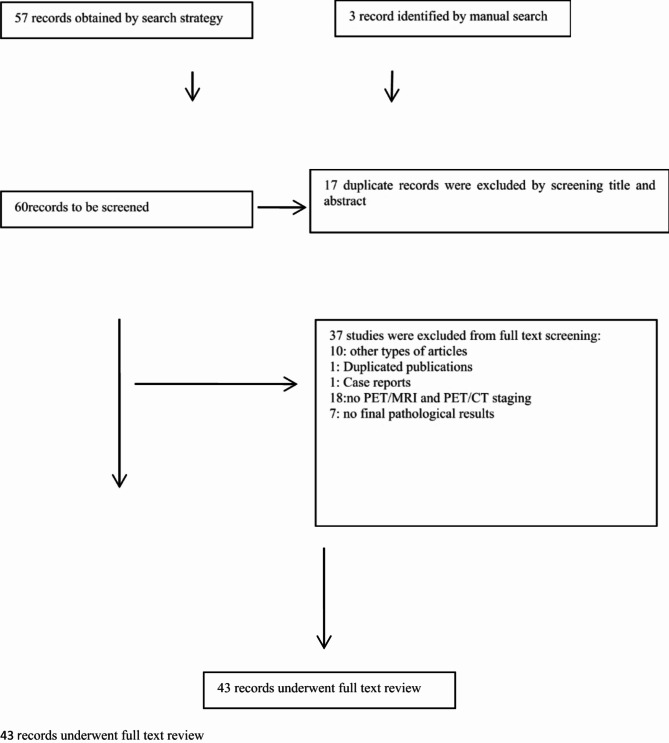
Flow diagram

As shown in the flow diagram (Fig. 1), 57 clinical studies were identified by search strategy, 3 studies were identified by manual search. And finally a total of 6 studies were finalized based on the predefined inclusion and exclusion criteria.

There were 123 patients in the 6 studies[6–7, 10–13], which all included PET/MRI and PET/CT. Among them, 5 studies compared PET/MRI staging, PET/CT staging, and pathological staging; 1 study compared PET/MRI with PET/CT staging but not compared pathological staging [13]. The study characteristics are shown in Table 1.

**Table 1:** The characteristics of studies

First author	Year	Country	No. of patients	Age	Radiopharmaceuticals	Pathological	PETMRI/CT Staging or not
Lee	2014	Korea	15	68.1 ± 7	^18^F-FDG-PET/MRI ^18^F-FDG-PET/CT	Oesophageal Cancer	Staging
Linder	2019	Sweden	16	65(46–78)	^18^F-FDG-PET/MRI ^18^F-FDG-PET/CT	Oesophageal and gastroesophageal junctional cancer	Staging
Liu	2019	China	26	34–76	^18^F-FDG-PET/MRI ^18^F-FDG-PET/CT	Gastric Cancer	Staging
Qin	2022	China	14	35–70	^68^Ga-DOTA-FAPI-04-PET/MR ^18^F-FDG-PET/CT	Gastric Cancer	Staging
Sharkey	2021	UK	22	68.8 ± 8.7	^18^F-FDG-PET/MRI ^18^F-FDG-PET/CT	Oesophageal/Gastro‑oesophageal cancer	Staging
Zheng	2020	China	30	34–76	^18^F-FDG-PET/MRI ^18^F-FDG-PET/CT	Gastric Cancer	No Staging

### Primary outcomes

Sensitivity in primary lesion detection.

**Fig. 2:**
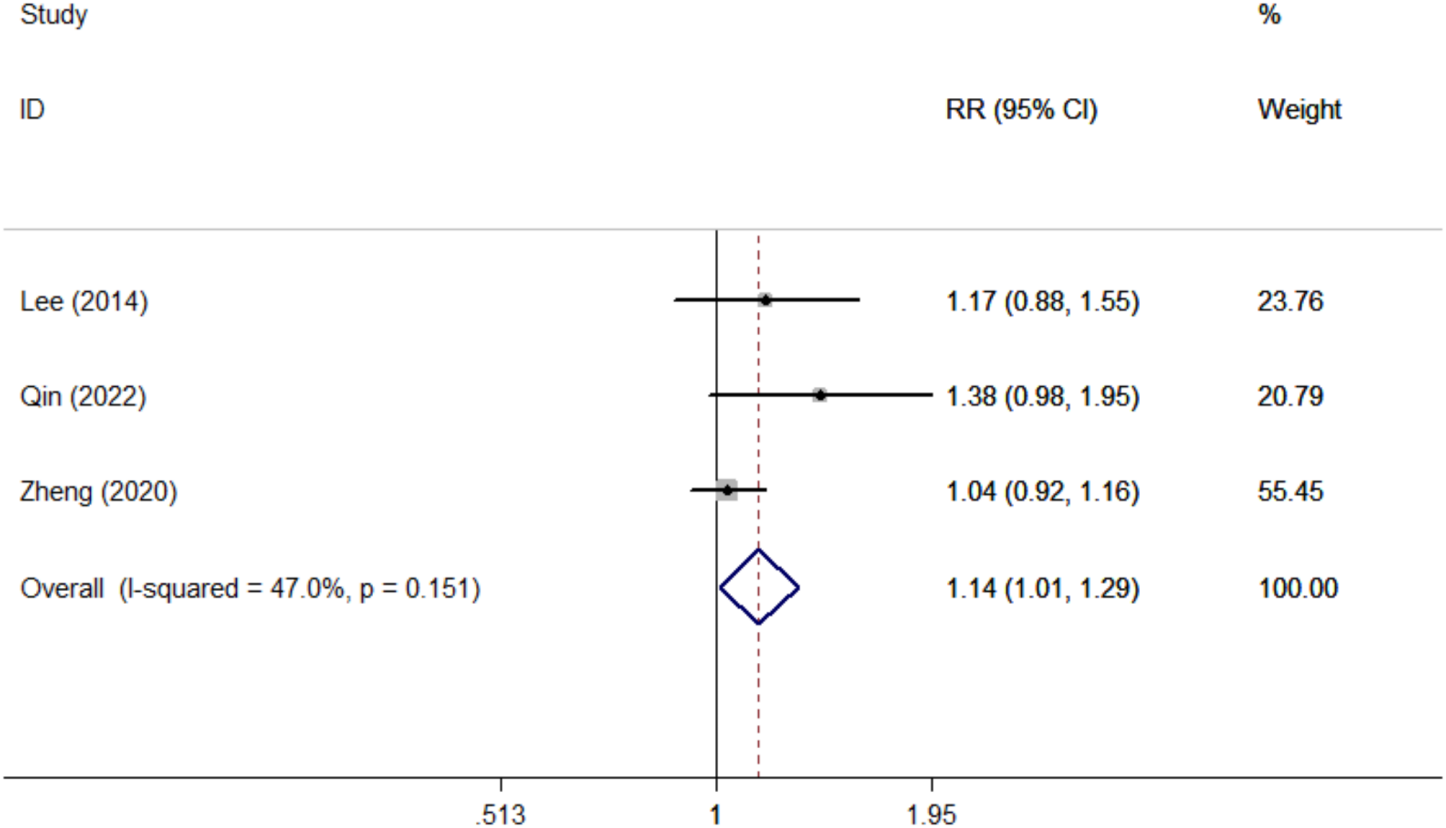
Forest plots of primary lesion detection evaluations

Three of the included studies reported the primary lesion detection evaluations(Table 2). PET/MRI had a comparatively high sensitivity in primary lesion detection evaluations compared with PET/CT. (RR = 1.14, 95% CI 1.01–1.29, *P* = 0.036) (Fig. 2).

**Table 2:** The characteristics of primary lesion detection evaluations

First author	Year	Country	No. of PET/MRI finding	No. of PET/CT finding	No. of Pathological finding
Lee	2014	Korea	14	12	15
Qin	2022	China	14	10	14
Zheng	2020	China	29	28	30

Accuracy of TNM staging.

**Fig. 3:**
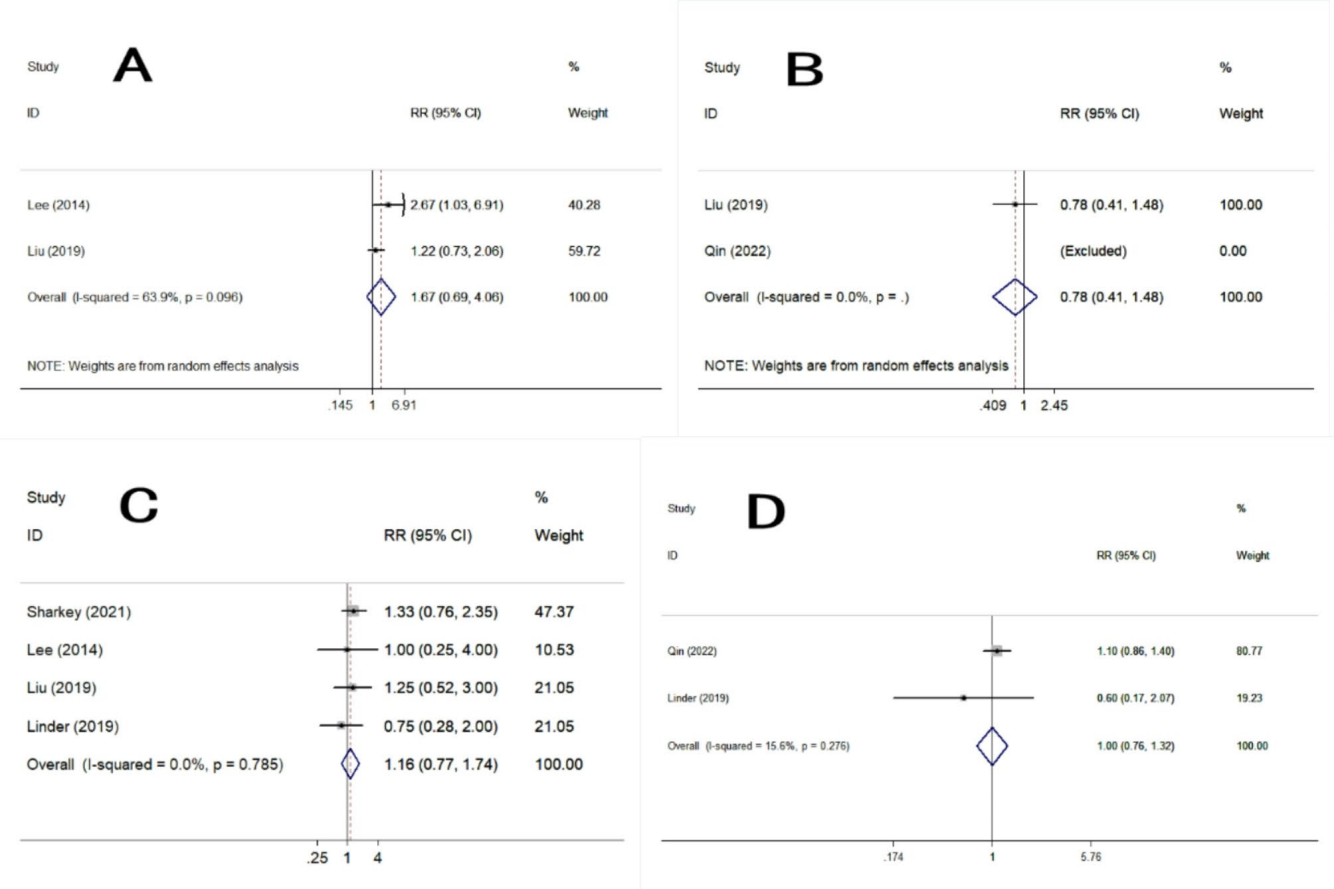
Forest plots of detection evaluations of T staging. **A**: T1. **B**: T2. **C**: T3. **D**: T4

**Fig. 4:**
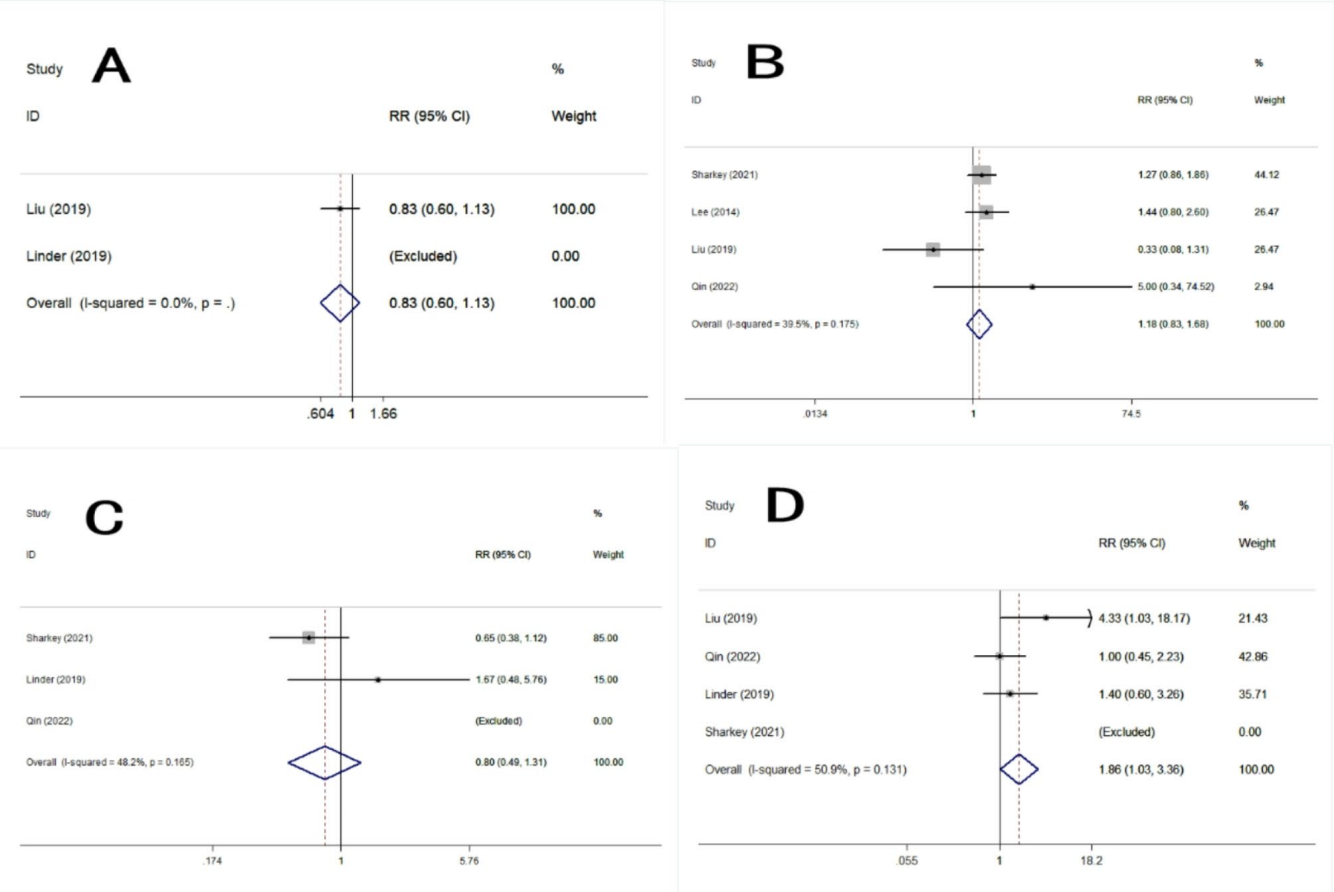
Forest plots of detection evaluations of N staging. **A**: N0. **B**: N1. **C**: N2. **D**: N3

**Fig. 5:**
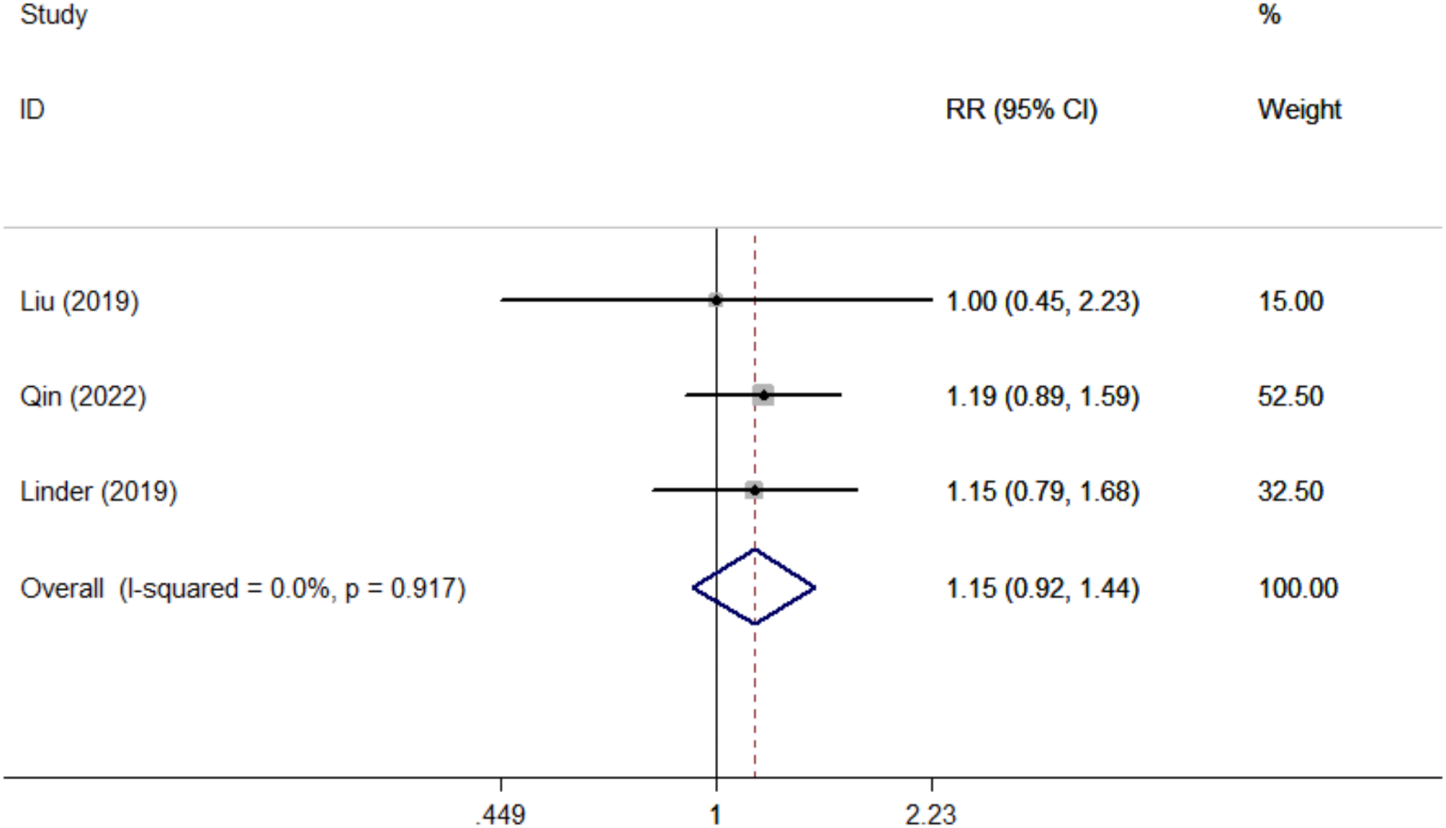
Forest plots of detection evaluations of M1 staging

Five studies reported the accuracy of TNM staging. PET/MRI had no significant statistical differences in all aspects of TNM staging compared with PET/CT: T1(RR = 1.67, 95% CI 0.69–4.06, *P* = 0.030), T2(RR = 0.78, 95% CI 0.41–1.48, *P* = 0.444), T3(RR = 1.16, 95% CI 0.77–1.74, *P* = 0.480), T4(RR = 1.00, 95% CI 0.76–1.32, *P* = 1.000), N0(RR = 0.83, 95% CI 0.60–1.13, *P* = 0.232), N1(RR = 1.18, 95% CI 0.83–1.68, *P* = 0.368), N2(RR = 0.80, 95% CI 0.49–1.31, *P* = 0.377), N3(RR = 1.86, 95% CI 1.03–3.36, *P* = 0.041), M1(RR = 1.15, 95% CI 0.92–1.44, *P* = 0.226).(Table 3, 4; Figs. 3, 4 and 5).

**Table 3:** The detection evaluations of T staging

First author	No. of PET/MRI finding				No. of PET/CT finding				No. of Pathological finding			
	T1	T2	T3	T4	T1	T2	T3	T4	T1	T2	T3	T4
Lee	8	4	2	0	3	7	2	0	9	2	4	0
Linder	0	12	3	1	0	8	4	4	0	8	6	2
Liu	5	3	5	13	4	6	4	12	5	4	8	8
Qin	0	0	3	11	0	0	0	10	0	1	2	11
Sharkey	0	5	12	5	0	12	9	1	0	0	18	4

**Table 4:** The detection evaluations of N and M staging

First author	No. of PET/MRI finding					No. of PET/CT finding					No. of Pathological finding					
	N0	N1	N2	N3	M1	N0	N1	N2	N3	M1	N0	N1	N2	N3	M1	
Lee	9	6	0	0	0	11	4	0	0	0	9	6	0	0	0	
Linder	5	6	2	3	7	5	8	1	2	6	5	6	2	3	7	
Liu	9	1	6	10	3	12	5	8	1	3	11	4	5	6	4	
Qin	5	2	4	3	12	7	0	4	3	10	3	3	4	4	12	
Sharkey	3	9	5	5	10	3	7	8	3	10	2	9	8	3	9	

## Discussion

In recent years, as PET/MRI has been gradually used in clinical, its advantages, such as higher soft tissue contrast and no risk of radiation exposure, have been gradually recognized compared to PET/CT. Now PET/MRI has been gradually used in various diseases throughout the body detection and staging of tumors [14]. However, previous studies have shown that the application of PET/MRI in gastric cancer and esophageal cancer is limited due to motion artifact interference caused by respiratory movement or gastrointestinal motility [15]. In recent years, with the continuous updating of MRI scanning technology, such as respiratory gating technology, studies have shown that PET/MRI is better than PET/CT in detecting gastric cancer and esophageal cancer [5, 13, 16]. In this study, the detection rate of PET/MRI for primary tumors of gastric and esophageal cancer was greater than that of PET/CT(*P* < 0.05), which is consistent with previous studies.

In terms of tumor T staging, most previous studies believe that PET/MRI is equivalent to PET/CT [14, 17]. Some studies even believe that PET/MRI is more accurate than PET/CT. They believe that PET/MRI has better soft tissue resolution, which can more clearly display the structure of each layer of the esophagus and gastric wall as well as the relationship between the tumor and surrounding tissues, and the display of the details of the tumor itself (such as shape, edges, etc.) is also better than PET/CT, so that it can be performed more accurately T staging [18–20]. In this study the results of Table 3 showed that PET/MRI classified more T3 stages than PET/CT (conversely for T2 stages), this could be related to higher spatial resolution of MRI and soft-tissue definition, but the meta-analysis statistical results of Fig. 3 showed that the the difference between them were not statistically significant, so we believe that the accuracy of PET/MRI in T1-4 stages were equivalent to that of PET/CT. It is speculated that it may be related to the sample size, so larger studies are necessary to fully assess the benefit of PET/MRI in N staging of oesophago and gastric cancer.

In terms of tumor N staging, previous studies have reported that PET/MRI is more accurate than PET/CT in N staging. It is speculated that in addition to providing lymph node size and metabolic uptake, PET/MRI also has more parameters for lymph node classification and judgment of metastasis, such as DWI and ADC values [21]. In this study the results of Table 4 showed that the PET/MRI demonstrates more N3 stages than PET/CT (which classifies more N2 patients), especially in the study by Liu 2019,this could be related to lymph node morphological characteristics, or DWI or post-gadolinium contrast behaviour, that are interpreted as possible metastasis, and which are not apparent on PET/CT in case these lymph nodes are metabolically negative on PET imaging.But the meta-analysis statistical results of Fig. 4 showed that the the difference between them were not statistically significant, it is speculated that the reason may be that the judgment of lymph node metastasis by imaging examination is more complicated, not simply relying on size or metabolic uptake criteria [22], and although PET/MRI can provide more judgment parameters, there is no unified judgment standard for each parameter [23], so its judgment on lymph node metastasis needs to be further studied.

In terms of tumor M staging, the results of this study showed that both PET/MRI and PET/CT had higher accuracy in detecting distant metastasis of tumors, and the difference between them was not statistically significant. It is also due to the high accuracy provided by MRI and the high specificity provided by PET, which enable PET/MRI to detect almost all bone metastases and distant organ metastases [24], which is consistent with our study.

This study had limitations: (1) The overall sample size of this study was relatively small, and studies with larger sample sizes are needed in the future to further confirm the application value of PET/MRI in the detection and staging of gastric and esophageal cancer. (2) The different PET radiopharmaceuticals may cause deviations to the results in this study. Some classification studies have shown that the new [68 Ga]Ga-FAPI-04 contrast agent may have better gastrointestinal tumor-promoting properties than the traditional [18 F]-FDG [25, 26]. So the PET/MRI using [68 Ga]Ga-FAPI-04 in this study [11] may achieve a higher detection rate and more accurate staging of gastric and esophageal cancer, but unfortunately, there is only one article which cannot be included in the meta-analyzed, so in the next step we will also conduct comparative studies on PET/MRI or PET/CT using different PET radiopharmaceuticals. (3) At present, compared with PET/CT, PET/MRI still lacks an internationally certified standardized scanning protocol. In this analysis, PET/MRI scanning protocols were also different, and some studies [27]reported the scanning plan or sequence might influence the results in detecting primary lesions and lymph nodes, so standardized PET/MRI protocols are needed to promote the quality and consistency of PET/MRI across centers, also to help streamline examinations and limit acquisition times.

## Conclusion

This systematic review confirmed the advantage of PET/MRI in detecting oesophago and gastric carcinomas.Compared with PET/CT, it can reduce unnecessary radiation exposure and can be used in relevant patients without contraindications of MRI.

## Data Availability

No datasets were generated or analysed during the current study.

## Abbreviations

PET/CT: Positron emission/Computed tomography
PET/MRI: Positron emission/Magnetic resonance imaging
CNKI: China national knowledge infrastructure
PRISMA: The preferred reporting items for systematic reviews and meta-analyses
RCTs: Randomized controlled trial
DWI: Diffusion weighted imagin
ADC: Apparent diffusion coefficient
